# A randomized controlled multicenter trial of post-suicide attempt case management for the prevention of further attempts in Japan (ACTION-J)

**DOI:** 10.1186/1471-2458-9-364

**Published:** 2009-09-26

**Authors:** Yoshio Hirayasu, Chiaki Kawanishi, Naohiro Yonemoto, Naoki Ishizuka, Yoshiro Okubo, Akio Sakai, Toshifumi Kishimoto, Hitoshi Miyaoka, Kotaro Otsuka, Yoshito Kamijo, Yutaka Matsuoka, Toru Aruga

**Affiliations:** 1 Department of Psychiatry, Yokohama City University School of Medicine, 3-9 Fukuura, Kanazawa-ku, Yokohama 236-0004, Japan; 2 Department of Biostatistics, School of Public Health, Kyoto University, Yoshidakonoecho, Sakyo-ku, Kyoto 606-8501, Japan; 3 Division of Preventive Medicine, Department of Community Health and Medicine, Research Institute, International Medical Center of Japan, 1-21-1 Toyama, Shinjuku-ku, Tokyo 162-8655, Japan; 4 Department of Neuropsychiatry, Nippon Medical School Tokyo, 1-1-5 Sendagi, Bunkyo-ku, Tokyo 113-8602, Japan; 5 Department of Neuropsychiatry, Iwate Medical University, 19-1 Uchimaru, Morioka, Iwate 020-8505, Japan; 6 Department of Psychiatry, Nara Medical University, 840 Shijo-cho, Kashihara, Nara 634-8521, Japan; 7 Department of Psychiatry, Kitasato University School of Medicine, 1-15-1 Kitasato, Sagamihara, Kanagawa 228-8555, Japan; 8 Department of Emergency and Critical Care Medicine, Kitasato University School of Medicine, 1-15-1 Kitasato, Sagamihara, Kanagawa 228-8555, Japan; 9 Department of Adult Mental Health, National Institute of Mental Health, National Center of Neurology and Psychiatry, 4-1-1 Ogawa-Higashi-cho, Kodaira, Tokyo 187-8553, Japan; 10 Department of Emergency Medicine, Showa University School of Medicine, 1-5-8 Hatanodai, Shinagawa-ku, Tokyo 142-8555, Japan

## Abstract

**Background:**

A previous suicide attempt is a potent risk factor for suicide later on. Crisis intervention, psychiatric and psychosocial evaluation at emergency medical facilities, and follow-up care for suicide attempters are considered important components for suicide prevention. The Japanese Multimodal Intervention Trials for Suicide Prevention (J-MISP) includes a randomized, controlled, multicenter trial of post-suicide attempt case management for the prevention of further attempts (ACTION-J) to address the continuing increase in suicides in Japan. The primary aim of ACTION-J is to examine the effectiveness of an extensive intervention for suicide attempters in prevention of recurrent suicidal behavior, as compared with standard intervention. This paper describes the rationale and protocol of the ACTION-J trial.

**Methods/Design:**

In this clinical trial, case management intervention will be provided at 19 emergency medical facilities in Japan. After crisis intervention including psychiatric evaluation, psychosocial assessment, and psychological education, subjects will be randomly assigned to either a group receiving continuous case management or a control group receiving standard care. Suicidal ideation, depressive symptoms, and general health condition will be evaluated as secondary measures. The intervention was initiated in July 2006. By December, 2009, 842 subjects will be randomized. Subject follow-up will continue for 1.5 to 5 years.

**Discussion:**

Suicide is a complex phenomenon that encompasses multiple factors. Case management by multi-sector collaboration is needed. ACTION-J may provide valuable information on suicide attempters and may develop effective case management to reduce future risk for suicide attempters.

**Trial registration:**

UMIN Clinical Trials Registry number, UMIN000000444. ClinicalTrials.gov number, NCT00736918.

## Background

### A history of suicide attempt as a risk factor for suicide

Based on studies in Europe, North America, and Australasia, a previous suicide attempt is a key risk factor for completed suicide [1-3]. After a follow-up period of 1 year, 12% to 15% of repetitions of cases of self-harm or suicide attempt are non-fatal, whereas 0.8% to 2.6% are fatal. After a follow-up period of 9 years, 3% to 12% ended in completed suicide [4]. Given these statistics, intervention for suicide attempters is an important element to prevent suicide.

### Recent increase in suicides in Japan

For approximately two decades (from 1978 to 1997), the suicide rate in Japan has been between 17.0 and 21.0 per 100,000 people. In 1997, 24,931 suicides were reported in Japan. In 1998, a dramatic 1.35-fold increase in the number of suicides in Japan occurred, as 32,863 suicides were reported. Since 1998, suicide rates in Japan have been between 25.2 and 27.0 per 100,000 people. For 11 years, the annual number of suicides in Japan has remained over 30,000 [5]. According to statistics from the World Health Organization (WHO) compiled in 2007 concerning worldwide suicide rates, the suicide rate in Japan was the eighth highest in the world [6].

#### Recent preventive measures against suicide in Japan

"The Declaration of Suicide Prevention" was issued in 2002 in Japan by the Advisory Panel on Strategy for Suicide Prevention. Since 2002, various measures associated with suicide prevention have been implemented, such as publication of suicide prevention manuals for the work place and medical practitioners. However, the number of suicides has not yet declined significantly. Therefore, in 2005, an intensive deliberation on suicide prevention was held by the Health, Labour, and Welfare Committee in the House of Councillors, and "The Resolution on Urgent and Effective Promotion of Comprehensive Strategies for Suicide" was passed in July 2005.

Also in 2005, two research projects (Japanese Multimodal Intervention Trials for Suicide Prevention: J-MISP [7]) funded by The Japanese Ministry of Health, Labor and Welfare (JMHLW), were launched to develop effective strategies to prevent suicide. J-MISP consists of a community intervention trial of a multimodal suicide prevention program (NOCOMIT) [8] and a randomized controlled multicenter trial of post-suicide attempt case management to prevent further attempts (ACTION-J).

### Review of strategies of intervention for suicide attempters

Various studies on intervention for suicide attempters as well as systematic reviews of these studies have been reported [9-14]. Few randomized controlled trials that focused on intervention methods showed a significant decrease in the repetition rate for attempted suicide. Van Herringen and colleagues investigated the effects of various strategies to increase compliance with referrals for outpatient aftercare [9]. Twenty-one of 196 patients (10.7%) in the experimental group and 34 of 195 patients (17.4%) in the control group repeated their suicidal behavior. The odds ratio was 0.57 (95% CI: 0.32 to 1.02).

A summary of 5 studies comparing cognitive behavioral therapy with standard aftercare demonstrated an odds ratio of 0.70 (confidence interval, 0.45 to 1.11), indicating the effects on suicide prevention. A summary of 6 studies involving intensive outreach, brief inpatient treatment, and nursing care, as compared with standard care, produced the odds ratio of 0.83 (CI: 0.61 to 1.14) [12].

Small sample sizes in the primary studies selected for the systematic review resulted in a wide range of confidence intervals for the odds ratios. Fewer than 600 subjects in both the experimental and control groups participated in the 5 studies to evaluate cognitive behavioral therapy and the 6 studies to investigate the effects of outreach programs. Thus, the total number of subjects in these studies was under 1,200. In addition, the follow-up period after enrollment was only 6 to 12 months. Hawton and colleagues [11] and Gaynes and colleagues [13], noting the limitations of studies with too few subjects and too short a study period, emphasized the need for large trials at multiple sites in order to determine the benefits of interventions.

### Overall scheme of ACTION-J

The act of suicide is complex. Findings from previous psychological autopsy studies in other countries indicate that more than 80% of patients who completed suicide could be diagnosed with a psychiatric disorder [15,16]. Over 80% of highly lethal (incomplete) suicide attempters taken to emergency medical centers in Japan were diagnosed with axis I psychiatric disorders, according to the Diagnostic and Statistical Manual of Mental Disorders (DSM-IV) [17]. Proper psychiatric assessment and treatment of suicide attempters may be critical to suicide prevention.

Based on these findings, we chose to utilize emergency medical facilities as trial sites and designed an intervention trial involving close collaboration between emergency medicine and psychiatric medicine for management of suicide attempters with psychiatric disorders. We planned a large-scale, multisite study in Japan.

In this trial, case management is employed as an intervention method. Case management provides multi-dimensional and comprehensive care that has not been studied in previous research, and includes psychological education, follow-ups to increase compliance with referrals for outpatient treatment, individualized casework including coordination of use of social resources, and information technology-based services. Prevention of further suicide attempts will be compared between subjects in the experimental group who receive the specialized, case management care and subjects in the control group who receive standard care.

### Objective of this study

The objective of this study is to examine the effectiveness of a trial intervention to prevent recurrent suicidal behavior by suicide attempters in Japan, as compared with a control intervention. It is expected that the case management administered in this study will be effective to prevent recurrence of suicide attempts.

## Methods/Design

ACTION-J is an open, randomized, controlled, multicenter study which examines the effectiveness of a trial intervention for suicide attempters in Japan. The trial intervention involves the implementation of case management for suicide attempters transported and admitted to emergency medical facilities. The task schedule is presented in Table 1.

**Table 1:** Task schedule

	**during admission**	**at discharge**	**1 w after discharge**	**4 w**	**8 w**	**12 w**	**6 m**	**12 m**	**18 m**	**24 m**	**30 m**	**36 m**	**42 m**	**Interim/Final analysis**
Psychiatric diagnosis	◎													
Psychoeducation 1*	◎													
Informed consent	◎													
Enrollment/randomization	◎													
Input data at time of discharge		◎												
Case management (Psychodeucation 2**, others)	○		○	○	○	○	○	○	○	○	○	○	○	
Psychiatric evaluation	◎						◎		◎		◎		◎	
Event	Input content of the event (ie, recurrent suicidal behavior, adverse event) into the web system as occasions require													
Participant survival (or cause of death of the participant)														◎
Actions to critical situations	In both groups during the study as occasions require													
Reports of a serious adverse event	Prompt report to the director of the hospital and the study group management office in both groups as occasions require													

◎: implemented in both groups; ○: implemented only in experimental intervention group

*: Psychoeducation Program I to all participants in both groups

**: Psychoeducation Program II to their family members during hospitalization in the experimental group

w: week, m: month

### Organization

JMHLW selected the Japan Foundation for Neuroscience and Mental Health (JFNMH) as the primary institution responsible for J-MISP, in close collaboration with the National Center of Neurology and Psychiatry. The J-MISP administration office in JFNMH will organize overall administrative procedures regarding the operations of the ACTION-J study group. The office will also establish and operate the steering committee, central research ethics committee, study evaluation committee, and study progress control committee.

The ACTION-J study group will include 19 participating hospitals in Japan. The study group will comprise the following: the study group management office, each participating hospital, the steering committee, the principal statistician, the independent statistician, the intervention program committee, the event review committee, and the data management center for technical support.

Each participating hospital will have psychiatrists, emergency department physicians, case managers, and other personnel. In addition, one coordinator, either a psychiatrist or an emergency physician, will be assigned to each participating hospital. Other participating researchers in this study include experts in suicide prevention, nurses, clinical psychologists, psychiatric social workers, biostatisticians, epidemiologists, and coordinators of the data management center.

### Subjects

Subjects will include individuals who are admitted to emergency medical facilities in Japan, are evaluated by an emergency physician or a psychiatrist in the emergency department, and are diagnosed as having made a suicide attempt. Subjects must also meet the following inclusion criteria:

### Inclusion criteria

1) Subject is over 20 years old.

2) Subject has been diagnosed with a psychiatric disorder classified into DSM-IV axis I.

3) Subject has had suicidal intentions confirmed at least twice using the Suicide Intent Scale [18].

4) Subject is able to understand the description of the study and provide informed consent.

5) During hospitalization, subject is able to attend an interview and the *Psychoeducation Program I *(see *Intervention *section), which will be required before enrollment in the study.

6) Subject is able to visit the participating hospital regularly for evaluations and case management and be contacted directly from the hospital on a regular basis.

### Exclusion criterion

1) Individual has a primary diagnosis that is not classified into DSM-IV axis I.

### Estimation of sample size

The total sample size is 842 participants, including 421 participants in each of the two treatment groups. Calculation of the desired sample size was based on the following rationale. According to a study of suicidal individuals transported to psychiatric emergency facilities in Japan, the annual incidence rate of events (including death) was set at 15% in the control group [19]. The target reduction in recurrent suicidal behavior in the trial intervention group was set at approximately 30%; the annual incidence rate of events (including death) in the intervention group was estimated to be 10.5% [20].

Based on this estimation, we calculated the sample size using the method of Shoenfeld and Richter, in order to confirm that the intervention group is superior, with a significance level of 2.5% for the one-sided test and a power of 90%, dependent on a 3.5-year-enrollment period and a 1.5-year follow-up period after enrollment. Given these assumptions, the desired number of participants per group was calculated to be 518, and number of events was expected to be 296. Sample size was set to increase the likelihood that the expected number of events (≥ 90% if no participant is lost to follow-up) would be observed during the study period.

### Informed consent

Participants will be patients admitted to the participating hospitals on an emergency basis, those who meet the inclusion criteria, and who provide informed consent to participate in this study.

### Enrollment

**Figure 1:**
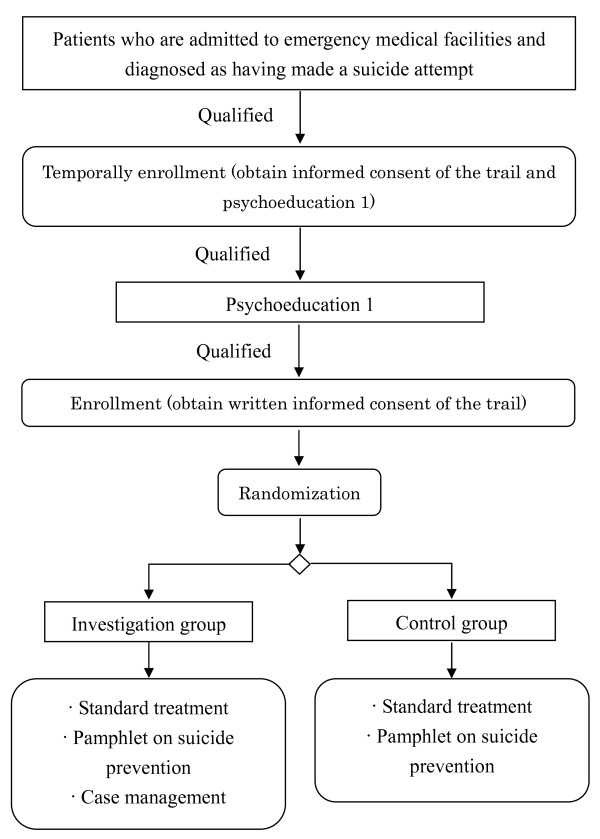
**Flow diagram of the study**.

Participant enrollment will be based on the following procedural outline (Figure 1). Any physician in an emergency facility will contact a psychiatrist when suspecting that a patient has made a suicide attempt. The psychiatrist will collect information and make a psychiatric diagnosis when examining the patient. At this point, the patient's suicidal intention will be confirmed (first check for suicidal intention). The investigator will confirm that the patient has not yet participated in this trial (i.e., that this event is not a repetition of suicidal behavior of a participant already enrolled in this trial) and will determine whether the patient is eligible to participate in this study by reviewing the inclusion and exclusion criteria. The investigator will explain this study, as well as the *Psychoeducation Program I *(see the description in the *Intervention section*), to a patient who is confirmed to have suicidal intentions and obtain patient consent. Next, a practitioner in charge of the psychoeducation program will provide the *Psychoeducation Program I *to the patient.

The investigator will again confirm the suicidal intentions of a patient who is deemed eligible to participate in the study. After the patient completes the *Psychoeducation Program I*, the investigator will reconfirm the suicidal intentions of a patient who is deemed eligible to participate in the study (second check for suicidal intention). The investigator will obtain written consent from the patient to participate in this study. On-site research staff at the participating hospital will collect data from the participant at the time of enrollment and enter the information via a web input system to receive a random assignment. The participant will be informed about his/her assigned group and the subsequent schedule within 1 week (by the time of the first interventional treatment).

### Randomization

Using the minimization method, participants will be randomly assigned to either the intervention group or control group. Central assignment involving an Internet-based assignment system will be performed.

Participants will be randomly assigned to one of the two groups according to the following factors:

1) Hospital

2) Gender

3) Age (< 40 or ≥ 40 years)

4) History of suicide attempts

### Intervention

All participants will attend the semi-structured *Psychoeducation Program I*, which will involve a discussion of psychological changes leading to suicide, risk factors for suicide and the relationship to psychiatric disorders; introduce stress management; demonstrate the usefulness of psychological and social support; and make patients aware of social resources. After randomization, the following interventions will be carried out in the respective groups (Table 1).

#### Case management intervention in the experimental group

Case managers will periodically contact participants assigned to the experimental intervention group (on the 1^st^, 4^th^, 8^th^, and 12^th ^week and the 6^th ^month after the day of written consent, and every 6 months thereafter until the end of the study). Case managers will inform participants about the date of their scheduled interviews in advance, via e-mail or regular mail. E-mail messages for participants will be prepared with the e-mail form on the input system and sent via the dedicated e-mail address for this study; the dedicated e-mail address does not permit any replies. Regular mail will be sent by participating hospitals, and words such as suicide will not be printed on the envelopes.

In principle, case management should be accomplished through direct dialogue (face-to-face interviews), where a telephone conversation is the next best option. Interviews should be conducted at participating hospitals. If case managers cannot reach participants, case managers will approach participant family members who have given their consent to be contacted in advance.

The interview scheduled for the first week should be conducted within two days before or after the scheduled date. Interviews for the 4^th^, 8^th^, and 12^th ^weeks should be conducted within a week, for the 6^th ^month within 2 weeks, and thereafter within 1 month before or after the scheduled date.

Case management will include the following activities:

1) Periodic interviews (either face-to-face or via telephone) with participants

2) Collection of information about each participant's background and treatment status

3) Encouragement of psychiatric treatment to the participants

4) Coordination of appointments with psychiatrists and primary care physicians

5) Encouragement of psychiatric treatment to the participants who have stopped receiving the treatment

6) Referrals to social resources and private support organizations and coordination for utilization of these resources

7) Providing information to participants and the *Psychoeducation Program II *to their family members during hospitalization

8) Providing Internet-based information (website only for the experimental intervention group)

Case managers will conduct periodic case conferences with psychiatrists. The study group management office and the intervention program committee will periodically hold case conference meetings with the study group, visit the participating hospitals, and meet with case managers, as necessary.

Regarding Internet-based information, participants in the experimental intervention group who access the website will receive information about the psychoeducation program, support organizations, and a self-diagnosis program. The dedicated intervention website will contain pages providing an introduction to social resources and serial articles, applied intervention (including psychoeducation and self-evaluation tools), and crisis intervention. The Intervention Program Committee will periodically update the content and articles on the website.

Standard treatment will be provided to subjects in the experimental group at each participating hospital. In addition, each participant in the experimental group will receive a pamphlet on suicide prevention following the psychoeducation program and at hospital visits after enrollment.

#### Control intervention

Participants in the control group will receive standard treatment with casework at the participating hospitals. Also, participants in the control group will receive a pamphlet on suicide prevention following the psychoeducation program and during their visits for periodic evaluations 6 months after enrollment and every year thereafter.

### Evaluations

#### Psychiatric Evaluations

Evaluators including psychiatrists, clinical psychologists, psychiatric social workers, and/or other mental health professionals, will conduct the psychiatric evaluations. In order to conduct blinded evaluations, evaluators will not know the participants' assigned groups, status of implementation of the intervention, or information on events obtained by other on-site research staff. Moreover, to achieve blinded evaluations, evaluators will not serve as case managers or practitioners in charge of the *Psychoeducation Program II*.

These evaluators will conduct psychiatric evaluations of all participants enrolled at the hospitals and will use a case sheet at 6 months from the date written consent was obtained and every year thereafter until the completion of the study. Evaluations can be carried out up to 1 month before or after the scheduled date.

Evaluations generally will take place as face-to-face interviews at the participating hospitals. The evaluators will notify the participants of the interview schedules 7 days before the scheduled dates via e-mail or regular mail. E-mail messages will be prepared with the e-mail form on the input system and sent via the dedicated e-mail address for this study; the dedicated email address does not permit any replies. Regular mail will be sent by the participating hospitals, and words such as suicide will not be printed on the envelopes. The evaluators will schedule the next evaluation date and inform participants at the end of each interview.

Evaluations will include the following:

1) Participant survival (or cause of death noted in the case of death of the participant)

2) Whether or not suicidal behavior has been repeated

3) Any events other than (1) or (2)

4) Stress factors

5) Persons and/or organizations to consult

6) Treatment status (outpatient or inpatient)

7) Physical function

8) Drinking habits

9) Evaluations using scales

a) Beck Hopelessness Scale [21]

b) Beck Depression Inventory-II (BDI-II) [22]

c) SF-36 [23]

#### Events

Events will be classified as follows:

1) Recurrent suicidal behavior

2) Total deaths (from any cause)

3) Self-harm

4) Adverse events other than (1), (2), or (3): Any unfavorable and unintended occurrence in a participant, whether or not there is a causal relationship with the intervention, will be recorded.

When identifying an event, the on-site research staff at the participating hospital will record the information according to the event review sheet and will confirm the information with the investigator. If there are no complications, the on-site research staff will enter the content of the event into the web input system. If necessary, on-site research staff will consult with the on-site research coordinator and the study group management office regarding any aspects of the event that are unclear. The on-site research coordinator will notify the hospital director about any serious adverse event and will fax the event review sheet directly to the study group management office.

The data center will consolidate the input data and periodically provide data to the study group management office and the chairperson of the event review committee, according to data management procedures. The study group management office and the chairperson of the event review committee should hold monthly event review meetings to evaluate and assess details of events based on the material provided.

Specific aspects of events will be described in the event definitions and event review procedures.

### Time periods during the study

Study period: August 2005 through March 2011

Enrollment period: July 2006 through December 2009

Follow-up period: July 2006 through June 2011

### Preconditions for hospital participation in the study

A hospital satisfying the following preconditions may participate in the study: The hospital should have both emergency medicine and psychiatry departments and an established collaborative agreement between those departments, so that the hospital can provide patients with psychiatric interventions to the emergency department.

Within the enrollment period, the hospital can recruit and obtain consent from at least 20 patients who are eligible to participate in the context of inclusion and exclusion criteria. The hospital will perform follow-up on the patients until study completion.

All participating researchers should take a seminar on suicide prevention (epidemiology, risk factors, psychology, prevention, intervention, and postvention). According to their respective roles, each participating researcher may take other seminars on psychiatric diagnosis (Mini International Neuropsychiatric Interview [M.I.N.I.; [24]]), the psychoeducation program, psychiatric evaluation, and assessment by scales (Suicide Intent Scale [18], Beck Hopelessness Scale [21], BDI-II [22], and SF-36 [23]).

### Approval of the study protocol

The study protocol will be reviewed and approved by the Central Research Ethics Committee. In principle, the study protocol also will have to be reviewed and approved by the On-site Research Ethics Committee at each participating hospital.

### Data collection

Data collection listed will be conducted according to the appropriate timing and each aspect of the relevant information.

#### Data collected at time of enrollment

1) Basic information on the participant

Initials, ID number, age, gender, other people living with the participant, marital status, education, employment, and other information

2) Information about suicidal behavior

Date and time, means, motivation, Beck Suicide Ideation Scale, and other details of past suicidal behavior

3) Demographic status (items marked with an asterisk on the forms are allocation adjustment factors): Age, gender, history of suicide attempts, DSM-IV diagnosis with M.I.N.I. [24], history of psychiatric treatment, history of hospital visits for physical problems, drinking habits, family history, and individuals to consult

4) Condition (psychiatric and physical diagnoses) at the time of enrollment

a) Suicide Intent Scale (only at the time of enrollment) [18]

b) Beck Hopelessness Scale [21]

c) BDI-II [22]

d) SF-36 [23]

#### Data collected at time of discharge

1) Date of hospital discharge

2) Discharge plans

#### Data collected during case management

1) Psychological and social conditions

2) Status of treatment for psychiatric and/or physical problems

3) Utilization of social resources

4) Utilization of dedicated intervention website

5) Degree of participant satisfaction with case management

#### Data collected during psychiatric evaluations

1) Participant survival (or cause of death of the participant)

2) Whether or not a suicidal behavior has been repeated

3) Any events other than (1) or (2)

4) Stress factors

5) Individuals and/or organizations to consult

6) Other medical services received (during clinical visits and/or hospital admission)

7) Physical function

8) Drinking habits

9) Evaluations using scales

a) Beck Hopelessness Scale [21]

b) BDI-II [22]

c) SF-36 [23]

### Outcomes

#### Primary outcome

The incidence of first recurrent suicidal behavior (expressed as attempted or completed suicides/person-year) will be used as the primary outcome, because an individual who reattempts suicide is at high risk for completion of suicide. Therefore, in order to develop effective suicide prevention strategies, it is essential to measure the time to the next suicidal behavior.

#### Secondary outcomes

Secondary outcomes will include the following:

1) Mortality rate (for any cause of death/person-year) during the study period2) The number and incidence rate of recurrent suicidal behavior, expressed as repeated suicidal attempts/person-year

3) The number of self-harm behaviors

4) Types and numbers of individuals and/or organizations to consult

5) Other medical services received (during clinical visits and/or hospital admission)

6) Physical function

7) Beck Hopelessness Scale score

8) BDI-II score

9) SF-36 score

### Evaluation of events

The event review committee will assess events related to the primary and secondary outcomes, while the assignment of the participants remains blinded. The event review committee will specify the evaluation criterion in the event definitions and event review procedures.

### Safety management

The on-site research staff at the participating hospitals will take necessary and appropriate actions to ensure the safety of participants when a serious adverse event occurs or a participant is at impending risk of suicide during the study. The on-site research staff will contact the on-site research coordinator at the hospital, and the coordinator will submit a report promptly to the director of the hospital and the study group management office.

### Statistical analyses

#### Primary analysis

The primary objective of this study is to examine whether or not the period of time until recurrent suicidal behavior (either attempt or completion of suicide) of participants in the experimental intervention group is significantly different from that of the control group. The stratified log-rank test based on allocation factors will be performed for all eligible participants in the intent-to-treat analysis, in order to examine the null hypothesis that the two groups are equal in the period of time until the incidence of the event.

A one-sided test will be conducted, because there would be no interest in the case that the experimental intervention is found to be significantly inferior to standard treatment. In this case, the level of significance will be set at 2.5% for the one-sided test, and the power will be set at 90%.

Sensitivity analyses will be performed as necessary, and a regression analysis will be performed with risk factors of potential influence.

#### Interim analyses

Interim analyses will be performed to evaluate achievement of the primary objective of the study. The analyses will be conducted twice during the study. Participant recruitment will be continued during the interim analyses.

For the interim analysis, the Lan-DeMets spending function will be used to adjust for multiplicity and to maintain the alpha error of the overall study at 2.5% for the one-sided test. The difference between the two groups in the period up to the event occurrence, using the O'Brien-Fleming alpha-spending function, will be examined for statistical significance.

The study will be terminated if the period up to the event occurrence in the trial intervention group exceeds that of the control group and the *p*-value of the log-rank sum test is less than the significance level defined by the method described above.

#### Secondary analysis

Secondary outcomes will be examined in order to reinforce the findings of the primary analysis. For analysis of secondary outcomes, the period up to event occurrence will be analyzed with the stratified log-rank test. Subgroup analysis of the primary and secondary outcomes will be performed by hospital, gender, age (< 40 or ≥ 40 years), and occurrence of suicide attempt before enrollment in this study. Because of the exploratory nature of the secondary analysis, no adjustment for multiplicity will be made.

### Ethical considerations

In this study, the rights and welfare of the participants will be protected according to the World Medical Association Declaration of Helsinki: Ethical Principles for Medical Research Involving Human Subjects. The study will comply with the ethical guidelines of the Ministry of Health, Labour, and Welfare in Japan. Ethical validity, including safety, scientific legitimacy, and reliability of results are ensured. Personal information collected by the participating hospitals in this study will include no identifiers that could be used to determine the identity of an individual and, therefore, will be made anonymous.

### Study monitoring

#### Periodic monitoring

The data management center will submit a monitoring report, including the information listed below, to the J-MISP administration office once every 3 months. The J-MISP administration office will send the monitoring reports to the study progress control committee, the Central Research Ethics Committee, and the study group management office.

The study progress control committee will examine the periodic monitoring reports and submit the evaluation to the J-MISP director. As a third party, the central research ethics committee will evaluate the periodic monitoring reports and make recommendations to the J-MISP director to revise the study protocol or stop the study, if ethical problems, such as safety and efficacy issues, should arise.

#### Contents of monitoring reports

1) Progress of the study, including enrollment

2) Status of the implementation of psychiatric evaluations

3) Data on the occurrence of events (according to the blinded group allocations)

4) Data on the occurrence of adverse events (according to the blinded group allocations)

5) Other relevant information, such as presence of undesirable issues and/or events

### Revision of the study protocol and due process

The J-MISP director will immediately inform the ACTION-J principal investigator of the decisions of the central research ethics committee if the committee has recommended that the study protocol be revised due to the emergence of safety issues based on the interim analysis, periodic monitoring, serious adverse events, and/or other issues that might affect the conduct of the study. The ACTION-J principal investigator will call a meeting of the study group and discuss protocol revision based on the decisions of the central research ethics committee. If necessary, the principal investigator will propose the revised study protocol and submit it to the J-MISP director.

If the study evaluation committee and the central research ethics committee approve the revised protocol, the J-MISP director will adopt the revised protocol after deliberation in the steering committee. The study group management office will immediately distribute the revised protocol to all the on-site research staff through the on-site research coordinator. The on-site research coordinator will submit the revised study protocol to the on-site research ethics committee at each participating hospital.

The study is to be resumed after the revised protocol has been approved by each committee.

### Study termination

Based on the findings of the interim analyses, the Central Research Ethics Committee can make a recommendation to the J-MISP director to terminate the study. The committee can decide to terminate the study because of safety issues. This decision will be based on the findings of the interim analyses, the periodic monitoring, the occurrence of a serious adverse event, or other issues that possibly could affect continuation of the study. The principal investigator will promptly convene a meeting of the steering committee to consider whether this study should be terminated, according to the conclusion of the central research ethics committee. Then, if study termination is confirmed to be appropriate, the final decision will be made.

## Discussion

Suicide is a complex phenomenon that encompasses multiple factors. Ratios between 10 and 18 suicide attempts to 1 completed suicide have been estimated in other countries [25,26]. Although the ratio of suicide attempts to completed suicide is not known in Japan, many patients with self-injury from suicide attempts are transported to emergency departments in Japan [5].

In the absence of effective measures against suicide attempters, it has been difficult to reverse an increasing suicide trend in Japan. Case management by multi-sector collaboration is required.

The ACTION-J study is designed to evaluate the effectiveness of emergency facility-based case management for suicide prevention in 19 participating hospitals in Japan. ACTION-J is intended to provide valuable information on suicide attempters and to develop effective case management to reduce future risk for suicide attempters.

## Competing interests

The authors declare that they have no competing interests.

## Authors' contributions

All authors participated in the design of the study. All authors contributed to the writing of the manuscript and have approved the final manuscript.

## Pre-publication history

The pre-publication history for this paper can be accessed here:


